# Utility of the Intradermal Skin Test in a Test-and-Cull Approach to Control Bovine Tuberculosis: A Pilot Study in Ethiopia

**DOI:** 10.3389/fvets.2022.823365

**Published:** 2022-03-07

**Authors:** Matios Lakew, Sreenidhi Srinivasan, Beruhtesfa Mesele, Abebe Olani, Tafesse Koran, Biniam Tadesse, Getnet Abie Mekonnen, Gizat Almaw, Temertu Sahlu, Bekele Seyoum, Kebede Beyecha, Balako Gumi, Gobena Ameni, Hagos Ashenafi, Douwe Bakker, Vivek Kapur, Solomon Gebre

**Affiliations:** ^1^National Animal Health Diagnostic and Investigation Center, Sebeta, Ethiopia; ^2^Aklilu Lemma Institute of Pathobiology, Addis Ababa University, Addis Ababa, Ethiopia; ^3^Huck Institutes of the Life Sciences, The Pennsylvania State University, University Park, PA, United States; ^4^Alage Agricultural Technical and Vocational Education Training (ATVET) College, Alage, Ethiopia; ^5^Department of Veterinary Medicine, College of Agriculture and Veterinary Medicine, United Arab Emirates University, Al Ain, United Arab Emirates; ^6^Independent Researcher and Technical Consultant, Lelystad, Netherlands; ^7^Department of Animal Science, The Pennsylvania State University, University Park, PA, United States

**Keywords:** bovine tuberculosis, control program, Ethiopia, prevalence, test and cull

## Abstract

Bovine tuberculosis (bTB) is one of the top three, high-priority, livestock diseases in Ethiopia and hence, the need for evaluation of potential control strategies is critical. Here, we applied the test-and-segregate followed by cull strategy for the control of bTB in the intensive Alage dairy farm in Ethiopia. All cattle reared on this farm were repeatedly skin tested using the Comparative Cervical Tuberculin (CCT) test for a total of five times between 2015 and 2021. During the first (October 2015) and second (March 2017) rounds of testing, all reactor animals (>4 mm) were culled, while those that were deemed as inconclusive (1–4 mm) were segregated and retested. At retest, animals with CCT >2 mm were removed from the herd. In the third (December 2017) and fourth (June 2018) rounds of tuberculin testing, a more stringent approach was taken wherein all reactors per the severe mode of CCT test interpretation (>2 mm) were culled. A final herd status check was performed in May 2021. In summary, the number of CCT positives (>4 mm) in the farm dropped from 23.1% (31/134) in October 2015 to 0% in December 2017 and remained 0% until May 2021. In contrast, the number of Single Cervical Tuberculin (SCT) test positives (≥4 mm) increased from 1.8 to 9.5% (from 2017 to 2021), indicating that CCT test might not be sufficient to effectively clear the herd of bTB. However, a more stringent approach would result in a drastic increase in the number of false positives. The total cost of the bTB control effort in this farm holding 134–200 cattle at any given time was conservatively estimated to be ~US$48,000. This, together with the need for culling an unacceptably high number of animals based on skin test status, makes the test-and-cull strategy impractical for nationwide implementation in Ethiopia and other low- and middle-income countries (LMICs) where the infection is endemic. Hence, there is an increased emphasis on the need to explore alternate, affordable measures such as vaccination alongside accurate diagnostics to help control bTB in endemic settings.

## Introduction

Bovine tuberculosis (bTB) is a chronic, progressive, granulomatous, inflammatory disease of cattle caused by members of the *Mycobacterium tuberculosis* complex (MTBC) (1). Most high-income countries have successfully controlled bTB based on test-and-slaughter of skin test-positive animals, alongside slaughterhouse surveillance, and trade and movement restrictions of affected herds. However, complete eradication is a challenge due to potential for spillover from wildlife reservoir hosts and imperfect performance of the currently available diagnostic tests (1–3). Conversely, in most low- and middle-income countries (LMICs), the bTB prevalence is high, and implementation of a test-and-slaughter program is not affordable for both social and economic reasons and, as a result, the disease has continued to cause significant economic and public health impacts (4). Moreover, the World Health Organization (WHO) estimates that of ~10 million new cases of human TB reported globally in 2018, ~71,000 to 240,000 were of zoonotic origin, meaning caused by *Mycobacterium bovis* alone (5). It is important to note that this is likely an underestimate due to the lack of cross-sectional, national-level surveillance programs, under-reporting and lack of laboratory confirmation of causative agents in LMICs where the infection is endemic in both human and bovine populations (5). Hence, it is being increasingly recognized that eliminating TB in humans cannot be accomplished without first controlling bTB in cattle.

A systematic review and meta-analysis conducted on the prevalence of bTB in Ethiopia reported a pooled prevalence estimate of 5.8% (95% CI: 4.5–7.5%) (6). The report indicated that bTB is highly prevalent in intensive and semi-intensive dairy farms in the country. However, so far there is no national bTB control strategy in Ethiopia, hence, this situation favors the unregulated movement of bTB-infected cattle and further spread of infection (7). Moreover, given the increased emphasis placed on expansion of intensive dairy farms with genetically improved dairy cattle to meet economic demands and nutritional requirements, the prevalence of bTB is only likely to worsen in the coming years. Hence, there is an urgent need for the implementation of a control program for bTB to reduce its prevalence in livestock and its zoonotic impact in Ethiopia (8, 9).

The tuberculin skin test is the current standard method for diagnosis of bTB recommended by the World Organization for Animal Health (OIE) (3). This test probes the cell-mediated immune response upon intradermal injection of the stimulating antigens such as purified protein derivatives (PPD) of tuberculin. In Ethiopia, the standard test for the diagnosis of bTB is the Comparative Cervical Tuberculin (CCT) test, which involves injection of two stimulating antigens, bovine PPD (PPD-B) derived from the extract of the *M. bovis* AN5 strain and avian PPD (PPD-A) derived from the extract of *M. avium* subsp. *avium* D4ER. The CCT is used to improve test specificity, although at the cost of sensitivity when compared with the use of the Single Cervical Tuberculin (SCT) test applying PPD-B only.

Here, we describe efforts involved in controlling the prevalence of bTB in a high burden voluntary dairy farm, owned by Alage Agricultural Technical and Vocational Education Training (ATVET) College in Ethiopia. An iterative strategy of repeated test-and-segregation followed by culling of reactors was implemented and followed for a period of 6 years. We also conservatively estimated the costs associated with this program.

## Materials and Methods

### Study Location

The study was conducted from 2015 to 2021 at the dairy farm of Alage ATVET College. This college occupies a total area of 4,200 ha, located at longitude of 38°30′ east and latitude of 07°30′ north. The college is situated about 217 km southwest of the capital city, Addis Ababa. It is part of the dry plateau agro-ecology of the southwestern part of the Ethiopian Rift Valley system at an altitude of 1,590 m above sea level. This dairy farm was established in 1979 with a starting population of 300 Holstein Friesian (HF) heifers from different sources and all animals have been exclusively stall-fed under a closed intensive management system. As part of farm biosecurity measures, new exotic breed animals have not been included since establishment. However, local Boran breed cattle are introduced for cross breeding purposes after screening for bTB. The farm is not in close proximity with any other dairy farms or wildlife populations. During the final round herd test (May 2021), the farm had a total of 175 cattle, of which 136 were HF-zebu cross, 33 were local Boran breed and 6 were calves <3 months of age. The dairy farm uses artificial insemination system for breeding and has its own veterinary clinic for animal health issues.

### Study Design

As part of our efforts to clear bTB from the Alage dairy farm, a repeated cross-sectional study was conducted whereby all animals (with the exception of calves <3 months of age, third trimester pregnancy (>8 months) and sick animals not suggestive of bTB), were skin tested with PPD-B and PPD-A for a total of five times between 2015 and 2021. The first round of tests was conducted in October 2015, the second in March 2017, the third in December 2017, and the fourth in June 2018. Finally, the current status of the farm was checked more recently in May 2021 using both CCT test and interferon-gamma release assay (IGRA). The testing interval between each round was not uniform but at least a 6 month gap was considered between rounds for logistical reasons. In the first and second rounds of testing, all animals testing positive (PPD-B–PPD-A > 4 mm) were slaughtered, while those in the inconclusive range (PPD-B–PPD-A = 1–4 mm) were segregated to a different location (~1 km away from the main herd with independent management) and retested 42 days later. At retest, animals with skin thickness >2 mm were slaughtered, and non-reactors were reintroduced into the main herd. Animals with skin thickness ranging from 1 to 2 mm at retest were kept segregated (until the next round of testing) instead of slaughter. Here, we note that this deviates from the OIE guidelines for retesting that recommends culling of all non-negative animals (≥1 mm) (3). However, including animals with skin thickness ranging from 1 to 2 mm dramatically increased the total number of animals to be culled and was not practically implementable in this dairy farm. In the third and fourth rounds of CCT testing, a more stringent approach was followed wherein all the tested animals with increase in skin thickness of >2 mm by CCT test were slaughtered and no retesting was conducted. Animals that showed 1–2 mm increase in skin thickness during the third round of test were segregated until fourth round test, while those from the fourth round remained in the herd. Three years later, the farm was tested again (May 2021) with CCT test and IGRA. It was recommended to the farm to segregate all animals that were inconclusive (1–4 mm) per CCT test, reactor (≥ 4 mm) per SCT test, and positive per IGRA (optical density ≥ 0.1).

### Skin Test Procedures

The CCT test was performed following the OIE guidelines (3, 10). Bovine PPD (3000IU/dose) and avian PPD (2500IU/dose) were administered intradermally with the bevel edge of needle faced outwards (0.1 ml final volume; Prionics/ThermoFisher Scientific, Lelystad B.V., The Netherlands). The same operator measured the skin thickness just before and 72 ± 4 h after the PPD injection. The difference in the increase of skin thickness at the PPD-B and PPD-A sites before and after injection was calculated. A reaction was considered positive if the increase in skin thickness at PPD-B site of injection was more than 4 mm greater than the reaction shown at the site of the PPD-A injection. The reaction was considered inconclusive if the PPD-B minus PPD-A reaction was from 1 to 4 mm, and negative if the PPD-B minus PPD-A reaction was <1 mm (3). During the third and fourth rounds of testing, all animals with skin thickness >2 mm by CCT test were considered positive per the severe mode of interpretation (11). In the SCT test (which involves PPD-B only), the reaction was considered positive if there is an increase of 4 mm or above in skin-fold thickness and inconclusive if the increase in skin-fold thickness is more than 2 mm and <4 mm. However, SCT results were not used for decisions on culling of animals.

### Interferon Gamma Release Assay

The IGRA test was conducted using the commercially available Gamma interferon test kit for Cattle (BOVIGAM^TM^, Prionics). Whole blood samples were collected using lithium heparin vacutainer tubes. The blood samples were stimulated with PPD-B and PPD-A, at a final concentration of 300 and 250 IU/ml, respectively, and were incubated in a humidified atmosphere at 37°C and 5% CO2 for 16–24 h. In addition, RPMI1640 media with L-Glutamine (BioWhittaker, Lonza, USA) and Pokeweed Mitogen (PWM) (Sigma-Aldrich) at 10 ug/ml final concentration were also used to stimulate the whole blood as negative and positive controls, respectively. Following overnight incubation, plates were centrifuged at 300 g for 10 min at room temperature and the supernatant was harvested. The harvested plasma was tested using the BOVIGAM ELISA kit to detect the secretion of interferon gamma (IFN-γ) from the stimulated *T* cells (12–14). Procedure was carried out as per kit instructions and the absorbance was measured at 450 nm with an ELISA reader. A reaction was considered positive if the optical density (OD) value of avian PPD subtracted from bovine PPD is ≥0.1.

### Cost Estimation

The costs incurred for implementation of the test-and-slaughter program at the Alage dairy farm was calculated in order to gain some insights into the overall expenses should such a program be implemented at national level in Ethiopia. This included the expenses associated with repeated testing of the animals for five rounds, reagents and relevant consumables, loss to the farm associated with culling (price of culled animals estimated per market price), personnel per diem and staff transportation costs. The meat of carcasses was officially inspected and those approved for consumption were sold at a price of 40 ETB (US$0.9) / kg and hence the income gained from selling the meat was considered as salvage value and deducted from the total cost.

### Data Analysis

All statistical analyses were performed using Prism 7 (GraphPad Software, La Jolla, CA) and Statistical Product and Service Solutions (SPSS) version 20. The chi-square (χ^2^) test was used for comparing prevalence estimates and *P* < 0.05 was considered for significance.

## Results

### Repeated Testing of the Alage Dairy Farm

The first round of testing (*n* = 134; all HF-Zebu cross breeds) was carried out in October of 2015 and the bTB within-herd prevalence was 23.1% (31/134) per the CCT test (skin thickness > 4 mm). All thirty-one reactor animals were culled, while animals presenting as inconclusive (1–4 mm; *n* = 30) were segregated in a different barn located 1 km away from the main Alage herd. Twenty-six (of the thirty) animals with inconclusive results were retested after 42-days of the first test, while the remaining four animals with inconclusive test results were exempted from the retesting as they were either under medical care or at late stages of pregnancy (Table 1).

**Table 1:** Summary of all skin tests conducted in the Alage dairy farm between 2015 and 2021.

**Test Date**	**Oct, 2015**	**March, 2017**	**December, 2017**	**June, 2018**	**May, 2021**
**Test round**	**1st**	**2nd**	**3rd**	**4th**	**5th**
**Number of animals tested**	134	173	169	181	169
**Test interpretation**	**Number of test positives (%)**				
CCT (PPDb-PPDa > 4)	31 (23.1)	8 (4.6)	0 (0.0)	0 (0.0)	0 (0.0)
CCT (PPDb-PPDa > 2)	46 (34.3)	15 (8.7)	1 (0.6)	4 (2.2)	2 (1.2)
CCT (PPDb-PPDa ≥ 1)	61 (45.5)	25 (14.45)	7 (4.1)	16 (8.8)	8 (4.7)
SCT (PPDb ≥ 4)	54 (40.3)	18 (10.4)	3 (1.8)	4 (2.2)	16 (9.5)
SCT (PPDb > 2)	93 (69.4)	36 (20.8)	8 (4.7)	25 (13.8)	23 (13.6)
Number culled	31	8	1	4	–
Number inconclusive (CCT, 1–4 mm)	30	17	7	16	8
Number retested	26	15	–	–	–
Number culled following retest	1	1	–	–	–

The second-round test (*n* = 173: 109 HF-Zebu cross and 64 Boran breed cattle) was conducted in March of 2017. The 64 local Boran cattle were not tested in the previous round and a decision was made to include them in this round of testing as they were managed in close proximity from the main Alage herd and to avoid any resulting spillover of infection. The prevalence of bTB in this round was found to be 4.6% (8/173), which was significantly lower than the prevalence observed in the first round (χ^2^ = 23.3; *P* < 0.0001). It is important to note that all the skin test-positive animals were among the newly included local Boran cattle. Seventeen animals (10 HF cross and 7 local Boran breed) showed inconclusive test results; 15 of them were retested and one (local Boran breed) showed positive skin reaction to the CCT test (Table 1). The reactor animal was culled, while the other animals were returned to the main Alage farm.

During the third-round test conducted in December 2017 (*n* = 169), there were no reactor animals per the CCT test (>4 mm) but 7 (4.1%) animals were inconclusive (1–4 mm). When severe mode of interpretation (>2 mm) was employed, one (HF cross breed) animal tested positive to the CCT test and it was slaughtered. The inconclusive animals were segregated from the main herd until the next round of testing. Compared to the prevalence in the second round of testing, the prevalence in the third round decreased significantly (χ^2^ = 7.936; *P* = 0.0048) as well.

The fourth-round test was carried out in June of 2018 (*n* = 181) and the CCT test result showed no bTB reactor animal in the herd per the > 4 mm interpretation. There were, however, 16 (8.8%) animals (5 HF cross and 11 local Boran cattle) that were classified as inconclusive per the CCT test (Table 1), of which four animals were slaughtered as they were positive by the severe mode of interpretation.

In order to evaluate the success of the control program implemented on the Alage farm from 2015 to 2018, the National Animal Health Diagnostic and Investigation Center (NAHDIC) team returned to the farm 3 years later in May of 2021 to conduct a fifth round of testing (*n* = 169). Both skin test and IGRA were performed. Encouragingly, there were no reactors identified per the CCT test interpretation of > 4 mm. However, 8 (4.7%) animals (7HF and 1 local Boran breed cattle) were inconclusive (1–4 mm). Moreover, 2 animals were categorized as reactors by the severe mode of interpretation (>2 mm). Per the SCT test, 16 animals were test-positive (≥ 4 mm).

**Figure 1:**
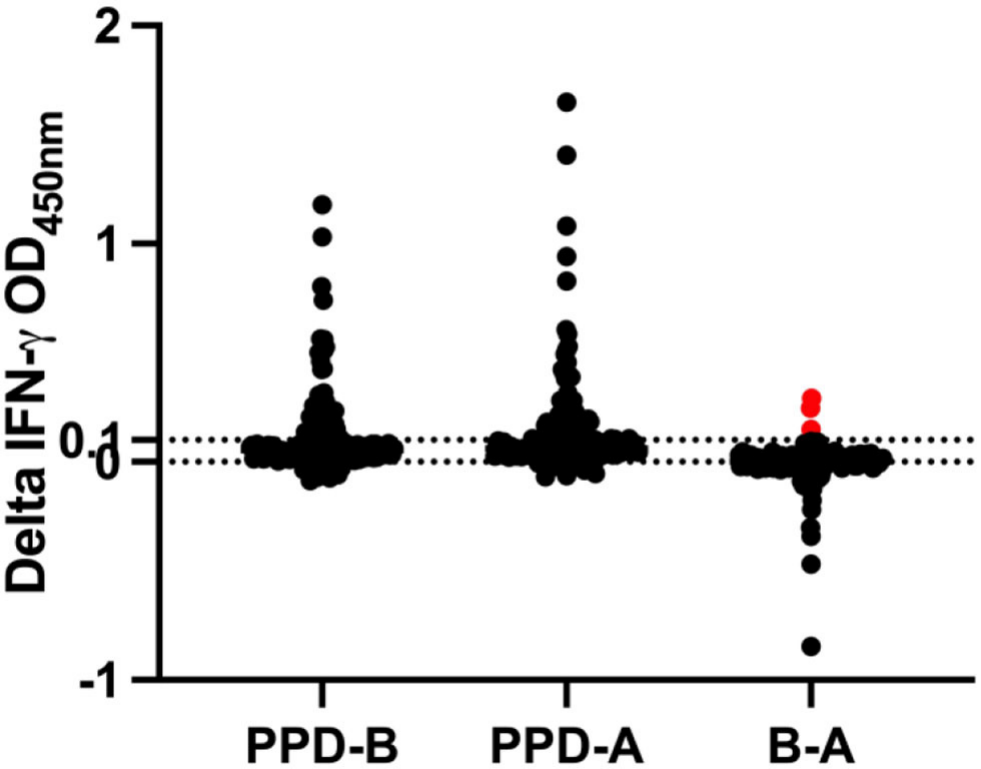
IGRA result during fifth-round test. Samples are positive if the OD value of PPD-A deducted from PPD-B is ≥0.1. The horizontal line at 0.1 denotes the cut-off. The red data points on B-A cross the cut-off of 0.1 and hence are reactors by IGRA.

In the IGRA, three animals had crossed the cut-off (optical density ≥0.1) (Figure 1). One of these three IGRA-positive animals was also positive per the severe mode of interpretation of CCT, while the other two are either positive or inconclusive per the SCT test. All animals that were inconclusive on CCT (1–4 mm), reactor on CCT per severe interpretation (>2 mm), reactor per SCT (≥ 4 mm) test and IGRA (optical density ≥ 0.1) were segregated from the herd (*n* = 21).

**Figure 2:**
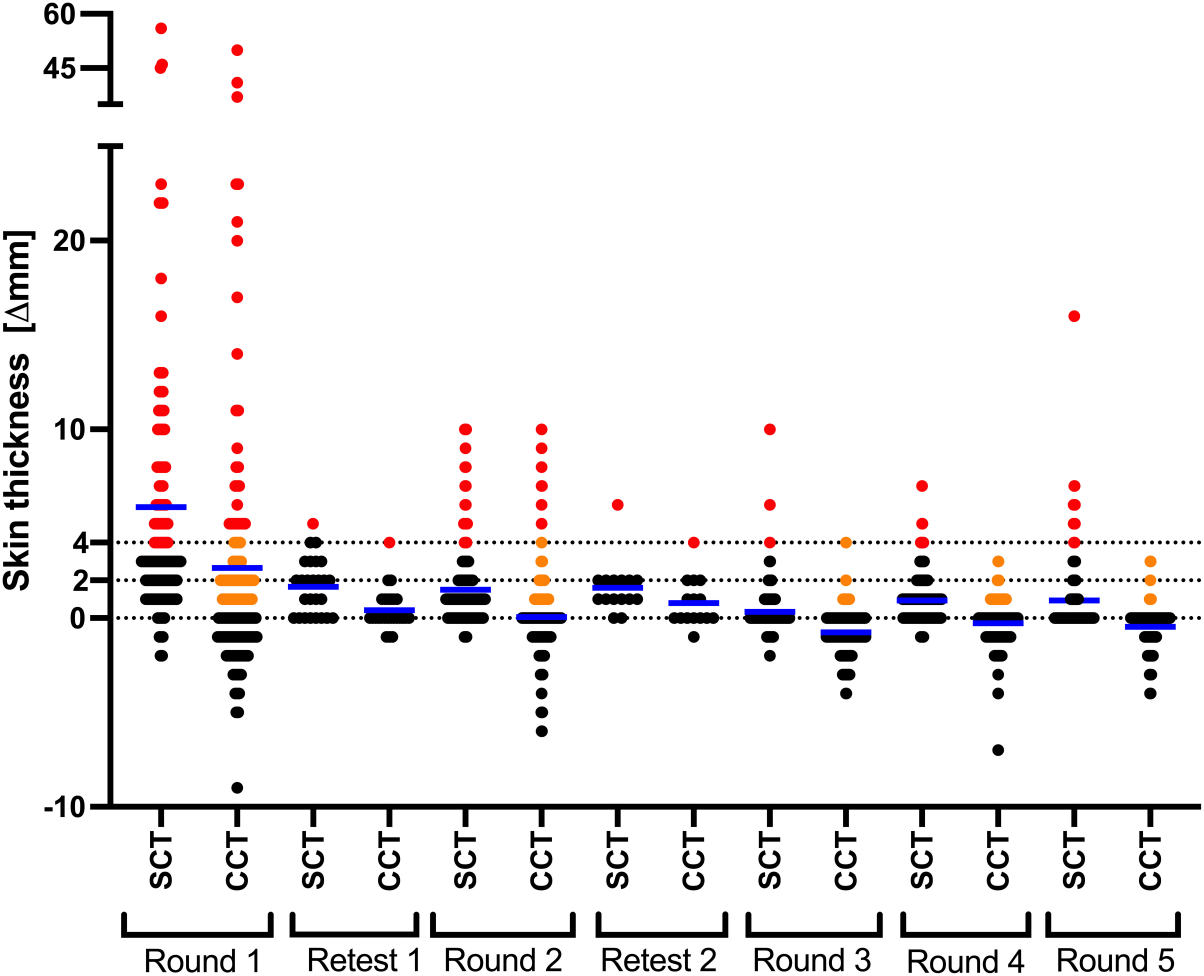
CCT and SCT test responses in every round of testing. Results are expressed as the difference in skin thickness (in millimeters) between the pre- and post-skin test readings, with the horizontal line providing the median (shown in blue). Black, orange and red data points represent animals that tested negative, inconclusive and positive, respectively. Retests in round 1 and 2 were conducted only on the animals in the doubtful range (orange).

Overall, following the repeated test-and-slaughter approach, the number of CCT positives (>4 mm) in the farm dropped from 23.1% (31/134) in October 2015 to 0% in December 2017 and remained 0% until May 2021. From Table 1, one can note that at all time points the number of test-positive animals per SCT (≥4 mm) was greater than that of CCT test, per both standard and severe interpretations (>4 mm and >2 mm). At the first testing round, the number of SCT test positives was 40.3%, which then dropped to 10.4% during the second round of testing. The lowest prevalence per SCT test was recorded during the third round (1.8%), after which it increased to 2.2% during the fourth round and then to 9.5% at the fifth-round test (Table 1 and Figure 2).

### Cost Analysis

The total cost of this program was conservatively estimated at ~US$ 48,000; the breakdown of each included expense item is provided in Table 2. The cost associated with skin testing alone, which includes cost of reagents and logistics for personnel was estimated to contribute ~18% to the total cost, while the major cost of the program (~82%) was associated with culling/slaughtering of reactor animals, i.e., estimated selling price of the culled/slaughtered animals. In total, 46 bTB reactor animals were slaughtered, of which 39 were conditionally approved for human consumption, and the salvage value of these animals was deducted from the total costs. However, the carcasses of the remaining seven animals were totally condemned with no salvage value (Table 2). All the expenditure of the fifth-round skin and IGRA test is also summarized and included to estimate the total cost.

**Table 2:** Conservative estimates of the cost associated with the bTB control efforts at Alage dairy farm.

**Category**	**Items**	**Details**	**Unit price (ETB)**	**Cost (ETB)**	**Cost (USD)**
**Skin testing**	Reagents	PPDs for 698 animals	100	69,800	2,094
		Tuberculin syringe	140	1,960	58
		Gloves, alcohol, blade and other consumables	246	3,400	102
	Personnel cost	4 people / 7 days for each test round	600 / day	100,800	3,024
		Fuel and travel (600 km per trip)	20 / liter	20,000	600
**Total**				195,960	5,878
**Handling of infected animals**	HF cross breed cow	33 culled	40,000	1,320,000	39,600
	Local breed cow	13 culled	15,000	195,000	5,850
	Cost to manage segregated animals	No new barn constructed; no new animal attendant employed			–
**Total**				1,515,000	45,450
**Total cost of the program (testing + culling of reactor animals)**				1,710,960	51,328
Salvage value	Meat recovered from 39 animals	~131.5 kg / animal	40 ETB / kg meat	205,140	6,154
**Total cost of fifth round skin and IGRA test**				122,740	2,860
**Grand total**				1,628,560	48,034

*Exchange rate used for estimating the cost of skin test from first to fourth round was 33.33 Ethiopian Birr (ETB) for one US$, based on the exchange rate dated April 27, 2020. Exchange rate used for estimating the cost of the fifth-round test was 42.9243 Ethiopian Birr (ETB) for one US$, based on the exchange rate dated May 21, 2021*.

## Discussion

Despite being a disease of economic and zoonotic importance, control measures for bTB are non-existent in Ethiopia and most other LMICs (4, 7). Earlier efforts to control bTB in a government farm in Ethiopia showed that upon repeated skin testing and segregation of skin test positive animals, the prevalence of bTB could be dramatically reduced from 48 to 1% in a dairy cattle herd (15). In the current study, NAHDIC in collaboration with Alage ATVET College implemented a bTB control strategy in the dairy farm affiliated with the college. The approach involved repeated tuberculin testing of the dairy herd with subsequent segregation and slaughter of test-positive animals from the farm. Accordingly, the entire cattle herd was tested four times between the years 2015 and 2018, with the exception of animals <3 months of age, animals >8 months pregnant, and those that were sick but not suggestive of bTB. All test-positive animals to the CCT test were removed, while animals categorized as inconclusive in the first and second rounds of testing were segregated and retested after 42 days. All positive animals in the retest were slaughtered. This test and segregate followed by retesting and slaughter approach over a 3-year period drastically decreased the within-herd prevalence of bTB from 23.1 to 0%, although a few animals that were identified as inconclusive per the CCT remained in the herd.

In order to check the status of the farm, the NAHDIC team returned to Alage 3 years later in May 2021. This time both skin test and IGRA were performed. Encouragingly, no animal was positive per the > 4 mm interpretation of CCT. However, there were a total of twenty-one animals that were reactors per SCT test and IGRA, and inconclusive per CCT test, that were segregated from the main herd. This is not surprising given the fact that the Alage farm was never made completely free of bTB as inconclusive animals identified in June 2018 continued to be a part of the farm. Starting from the third round (December 2017) to the fifth-round test (May 2021) all the animals were negative per the CCT > 4 mm interpretation, however the number of PPD-B reactors (SCT > 2 mm and ≥ 4 mm) did increase in the last testing rounds. This may indicate that CCT > 4 mm or even the severe interpretation CCT > 2 mm may not be sufficient in certifying previously bTB infected herds as bTB-free herds. Hence, further attention should be given to the development of alternative diagnostic reagents to reduce the number of false positives, uncertainties associated with the PPD skin test, and limit conflicts with herd owners. It is important to acknowledge here that bacteriological culture and confirmation of reactor animals could not be performed and is a limitation of the study. While the number of animals positive per SCT was high, it is also important to note the relative low specificity of this test, in particular in the context of regions with high environmental bacteria such as Ethiopia, further stressing the need for more accurate and specific defined diagnostic reagents (8, 10, 16). The increase in the number of reactor animals due to the interruption in routine testing between June 2018 and May 2021 indicates that expenses involved in the control of bTB are anything but short-term in nature. Despite having culled a total of 46 cattle, there were still some inconclusive animals in the final round, suggesting that extant tuberculin-based tests may not be sufficient tools for accomplishing disease eradication in endemic settings and new defined antigen tests are an urgent need (16).

As has been proven time and again, test-and-cull-based programs can be hugely successful in efficiently controlling bTB (17). The rigorous application of tuberculin testing and culling of reactors has significantly reduced the prevalence and even eliminated bTB infection from farmed bovine populations in many high-income countries in the past (3, 18–20). For example, in the Unites States (US), a test-and-cull-based control program was initiated over a century ago in 1917, and together with introduction of milk pasteurization, this control program has been regarded as one of the most successful campaigns ever waged against a bacterial disease. Between 1917 and 1940, ~232 million cattle were screened, of which, ~3.8 million were culled. Documented reports show a 10-fold return on investment in lives saved and economic benefits to farmers in the US (21). However, can such a test and cull approach be practically implemented at a national level in Ethiopia and other LMICs? While the results of this study in terms of reduction in disease prevalence are encouraging, conservative cost estimates suggest that the total expenditure during the 3-year effort and the final fifth round test in a farm holding <200 cattle was ~US$48,000. It is important to note that this cost estimate does not include the milk losses associated with culling of lactating cows, possible meat productivity loss, additional housing and management costs to segregate test-inconclusive animals, and the cost of social and zoonotic impacts. The national priorities for intensification of dairy production in countries like Ethiopia will only worsen the disease burden resulting in further increase in economic losses due to bTB. Ethiopia has the largest cattle population in Africa of ~61.5 million heads (22) and applying a conservative pooled prevalence estimate of 5.8% (95% CI: 4.5 to 7.5%) as reported in a recent meta-analysis, there may be ~3.5 million bTB-infected cattle in the country. While we acknowledge that additional studies are required to accurately predict the costs and benefits of implementing a national level test and cull-based control strategy, it is quite apparent that a program of such a scale is far from feasible in Ethiopia and other LMICs for both economic and social reasons. Moreover, there are multiple complications in adapting this approach in non-dairy herds and pastoral production systems given extensive management styles and cultural reasons.

In this context, it is important to highlight the need for a vaccine that can reduce the burden of infection and transmission. Recent reports including natural transmission studies conducted in endemic settings have shown that Bacillus Calmette-Guérin (BCG) vaccination may have considerable utility in this regard (23). While BCG has been used for experimental vaccination of cattle against bTB since 1913, it is not yet licensed for field use as it sensitizes animals to the tuberculin-based skin tests (3, 24). This compromises the specificity of the skin test and results in an inability to differentiate infected from vaccinated animals (DIVA). Therefore, bTB control programs that use the OIE-prescribed tuberculin skin test also prohibit the use of BCG vaccination. However, safety and BCG-induced protection (although partial) of cattle against experimental challenge with *M. bovis* has been known since the promising early reports published by Calmette and Guérin. A recent meta-analysis that attempted to synthesize data from published studies across the globe estimated a direct BCG vaccine efficacy of 25% (95% CI: 18, 32) (25). While no study has yet rigorously assessed indirect vaccine efficacy, a significant reduction in lesion severity observed following BCG vaccination likely contributes to reduction in risk of transmission from vaccinates to susceptible cattle (indirect efficacy). It was also emphasized through scenario analyses with transmission dynamic models incorporating direct and indirect vaccinal effects (“herd-immunity”), that BCG vaccination alongside a DIVA diagnostic test appears to be the most promising option in endemic settings in the near future. However, this warrants detailed cost-benefit analyses prior to rolling out BCG vaccination in endemic settings. In this context, progress has been made in the field of bTB diagnosis in the development and validation of peptide-based skin test reagents comprising of antigens that are present in pathogenic strains of MTBC and absent in the vaccine strain, BCG (16, 26). The DIVA capability of these antigens has also been successfully demonstrated in both experimental and naturally infected animals, hence enabling the use of BCG vaccination as part of future bTB control programs (27, 28). It is important to note here the ease of chemical synthesis, quality control and cost-effectiveness of these peptide-based reagents.

Taken together, while the repeated test and cull approach is now being implemented at some voluntary government and private dairy farms in Ethiopia, it may not be a viable option at the national level for reasons discussed here. Hence, feasible alternatives need to be sought urgently to control bTB, an important step towards the WHO goal of ending the global tuberculosis epidemic by 2035 (29). Vaccination against bTB with BCG has been researched for over a century and provides strong evidence for the consideration of implementation of vaccine-based bTB control strategies, particularly in LMICs and other high burden settings. While the reported direct efficacy estimates are modest, it is important to note that indirect efficacy of BCG remains largely unexplored and that further investigations are needed to address this critical knowledge gap. Given the predicted intensification of cattle herds in endemic settings, these observations will have major implications for informing and implementing practical disease control policies in Ethiopia and other LMICs.

## Conclusion

This study showed that the test and cull program significantly reduced the prevalence of bTB from Alage ATVET dairy farm within 3 years. However, the cost estimation indicates that it may not be practical to scale-up and implement nationwide in Ethiopia and other LMICs. Hence, exploring feasible and economically affordable alternative bTB control options such as test-and-segregate approaches, and vaccination need priority.

## Data Availability Statement

The original contributions presented in the study are included in the article, further inquiries can be directed to the corresponding author.

## Ethics Statement

The animal study was reviewed and approved by National Research Ethics Review Committee-NRERC No. 3.10/800/07.

## Author Contributions

ML, BM, AO, TK, GAl, SG, TS, and KB conceived and designed the study. ML, BM, AO, TK, BS, and GAm conducted the research. ML, AO, BT, BG, GAl, HA, DB, VK, and SS analyzed the data, drafted the paper, and contributed to writing. All authors read and approved the final manuscript.

## Funding

All reagent and salary/per diem expenses were supported by NAHDIC's internal funds. The other expenditure associated with culling of reactor animals was covered by Alage Agricultural Technical and Vocational Education Training (ATVET) College. The cost for the fifth-round testing was covered by Accelerating Bovine Tuberculosis Control in Developing Countries (AB_TB_CD) project which is supported by a grant (OPP1176950) from the Bill & Melinda Gates Foundation and the U.K. Department for International Development to the Pennsylvania State University.

## Conflict of Interest

The authors declare that the research was conducted in the absence of any commercial or financial relationships that could be construed as a potential conflict of interest.

## Publisher's Note

All claims expressed in this article are solely those of the authors and do not necessarily represent those of their affiliated organizations, or those of the publisher, the editors and the reviewers. Any product that may be evaluated in this article, or claim that may be made by its manufacturer, is not guaranteed or endorsed by the publisher.

## Abbreviations

ATVET: Agricultural Technical and Vocational Education Training
BCG: Bacille Calmette-Guerin
bTB: bovine tuberculosis
IGRA: interferon gamma release assay
LMICs: low- and middle-income countries
OIE: World Organization for Animal Health
CCT: Comparative Cervical Tuberculin
MTBC: *Mycobacterium tuberculosis* complex
NAHDIC: National Animal Health Diagnostic and Investigation Center
SCT: Single Cervical Tuberculin
WHO: World Health Organization.

## References

[1] OIE. Bovine Tuberculosis, Information on Aquatic and Terrestrial Animal Diseases. (2019). Available online at: https://www.oie.int/en/animal-health-in-the-world/animal-diseases/bovine-tuberculosis/#F (accessed on April 2020).

[2] Napp S Ciaravino G de Val BP Casal J Saéz JL Alba A. Evaluation of the effectiveness of the surveillance system for tuberculosis in cattle in Spain. Prev Vet Med. (2019) 173:104805. 10.1016/j.prevetmed.2019.104805 31715496

[3] OIE. Bovine *tuberculosis, Office International des Epizooties Terrestrial Manual.* Chapter 3.4.6. (2018). pp. 1058–1074 (accessed May 25, 2020).

[4] WHO FAO OIE The-Union. Roadmap for zoonotic tuberculosis. World Health Organization (WHO), Food and Agriculture Organization of the United Nations (FAO), World Organisation for Animal Health (OIE) and the Union- International Union Against Tuberculosis and Lung Disease. (2017). Available online at: https://www.oie.int/fileadmin/Home/eng/Our_scientific_expertise/docs/pdf/Tuberculosis/Roadmap_zoonotic_TB.pdf (accessed June 14, 2020).

[5] WHO. World Health Organization, Global Tuberculosis Report of 2019. (2019). Available online at: https://www.who.int/tb/publications/global_report/en/ (accessed January 15, 2021).

[6] Sibhat B Asmare K Demissie K Ayelet G Mamo G Ameni G. Bovine tuberculosis in Ethiopia: a systematic review and meta-analysis. Prev Vet Med. (2017) 147:149–57. 10.1016/j.prevetmed.2017.09.006 29254713 PMC5739073

[7] Areda DB Muwonge A Dibaba AB. Status of bovine tuberculosis in Ethiopia: challenges and opportunities for future control and prevention. In: Dibaba A Kriek N Thoen C, editors. Tuberculosis in Animals: An African Perspective. Cham: Springer (2019). p. 317–37. 10.1007/978-3-030-18690-6_14

[8] Firdessa R Tschopp R Wubete A Sombo M Hailu E Erenso G. High prevalence of bovine tuberculosis in dairy cattle in central Ethiopia: implications for the dairy industry and public health. PLoS One. (2012) 7:e52851. 10.1371/journal.pone.0052851 23285202 PMC3532161

[9] Mekonnen GA Conlan AJK Berg S Ayele BT Alemu A Guta S. Prevalence of bovine tuberculosis and its associated risk factors in the emerging dairy belts of regional cities in Ethiopia. Prev Vet Med. (2019) 168:81–9. 10.1016/j.prevetmed.2019.04.010 31097127 PMC10364076

[10] Middleton S Steinbach S Coad M McGill K Brady C Duignan A. A molecularly defined skin test reagent for the diagnosis of bovine tuberculosis compatible with vaccination against Johne's Disease. Sci Rep. (2021) 11:2929. 10.1038/s41598-021-82434-7 33536465 PMC7859399

[11] Ameni G Hewinson G Aseffa A Young D Vordermeier M. Appraisal of interpretation criteria for the comparative intradermal tuberculin test for diagnosis of tuberculosis in cattle in central Ethiopia. Clin Vaccine Immunol. (2008) 15:1272–6. 10.1128/CVI.00114-08 18495847 PMC2519295

[12] Wood PR Corner LA Plackett P. Development of a simple, rapid in vitro cellular assay for bovine tuberculosis based on the production of gamma interferon. Res Vet Sci. (1990) 49:46–9. 10.1016/S0034-5288(18)31044-0 2116655

[13] Wood PR Corner LA Rothel JS Baldock C Jones SL Cousins DB. Field comparison of the interferon-gamma assay and the intradermal tuberculin test for the diagnosis of bovine tuberculosis. Aust Vet J. (1991) 68:286–90. 10.1111/j.1751-0813.1991.tb03254.x 1953560

[14] OIE. BOVIGAM®-Mycobacterium bovis Gamma Interferon Test Kit for Cattle. OIE Procedure for Registration of Diagnostic Kits, Abstract sheet. (2015). Available online at: https://www.oie.int/app/uploads/2021/03/oie-register-bovigam-abstract-v1-05-2015.pdf (accessed March, 2021).

[15] Ameni G Aseffa A Sirak A Engers H Young DB Hewinson RG. Effect of skin testing and segregation on the prevalence of bovine tuberculosis, and molecular typing of Mycobacterium bovis, in Ethiopia. Vet Rec. (2007) 161:782–6. 18065813 PMC2292248

[16] Srinivasan S Jones G Veerasami M Steinbach S Holder T Zewude A. A defined antigen skin test for the diagnosis of bovine tuberculosis. Sci Adv. (2019) 5:eaax4899. 10.1126/sciadv.aax4899 31328169 PMC6636981

[17] Cousins DV. Mycobacterium bovis infection and control in domestic livestock. Rev Sci Tech. (2001) 20:71–85. 10.20506/rst.20.1.1263 11288521

[18] More SJ Radunz B Glanville RJ. Lessons learned during the successful eradication of bovine tuberculosis from Australia. Vet Rec. (2015) 177:224–32. 10.1136/vr.103163 26338937 PMC4602242

[19] Palmer MV Waters WR. Bovine tuberculosis and the establishment of an eradication program in the United States: role of veterinarians. Vet Med Int. (2011) 2011:816345. 10.4061/2011/816345 21647341 PMC3103864

[20] Reviriego Gordejo FJ Vermeersch JP. Towards eradication of bovine tuberculosis in the European Union. Vet Microbiol. (2006) 112:101–9. 10.1016/j.vetmic.2005.11.034 16388921

[21] Olmstead AL Rhode PW. An impossible undertaking: the eradication of bovine tuberculosis in the United States. J Econ Hist. (2004) 64:734–72. 10.1017/S0022050704002955

[22] CSA. Agricultural Sample Survey 2018/19, Report on Livestock and Livestock Characteristics, Volume II. Central Statistical Agency (2019).

[23] Ameni G Tafess K Zewde A Eguale T Tilahun M Hailu T. Vaccination of calves with Mycobacterium bovis Bacillus Calmette-Guerin reduces the frequency and severity of lesions of bovine tuberculosis under a natural transmission setting in Ethiopia. Transbound Emerg Dis. (2018) 65:96–104. 10.1111/tbed.12618 28168855 PMC5811905

[24] Luca S Mihaescu T. History of BCG Vaccine. Maedica. (2013) 8:53–8. 24023600 PMC3749764

[25] Srinivasan S Conlan AJK Easterling LA Herrera C Dandapat P Veerasami M. A meta-analysis of the effect of Bacillus Calmette-Guérin vaccination against bovine tuberculosis: is perfect the enemy of good? Front Vet Sci. (2021). 8:637580. 10.3389/fvets.2021.637580 33681334 PMC7930010

[26] Whelan AO Clifford D Upadhyay B Breadon EL McNair J Hewinson GR. Development of a skin test for bovine tuberculosis for differentiating infected from vaccinated animals. J Clin Microbiol. (2010) 48:3176–81. 10.1128/JCM.00420-10 20592155 PMC2937719

[27] Bayissa B Sirak A Zewude A Worku A Gumi B Berg S. Field evaluation of specific mycobacterial protein-based skin test for the differentiation of Mycobacterium bovis-infected and Bacillus Calmette Guerin-vaccinated crossbred cattle in Ethiopia. Transbound Emerg Dis. (2021). 10.1111/tbed.14252. [Epub ahead of print]. 34331511 PMC8801543

[28] Srinivasan S Subramanian S Shankar Balakrishnan S Ramaiyan Selvaraju K Manomohan V Selladurai S. A defined antigen skin test that enables implementation of BCG vaccination for control of bovine tuberculosis: proof of concept. Front Vet Sci. (2020) 7:391. 10.3389/fvets.2020.00391 32793643 PMC7393633

[29] Uplekar M Weil D Lonnroth K Jaramillo E Lienhardt C Dias HM. WHO's new end TB strategy. Lancet. (2015) 385:1799–801. 10.1016/S0140-6736(15)60570-0 25814376

